# Enhanced room temperature gas sensing properties of low temperature solution processed ZnO/CuO heterojunction

**DOI:** 10.1186/s13065-019-0519-5

**Published:** 2019-01-29

**Authors:** P. P. Subha, M. K. Jayaraj

**Affiliations:** Nanophotonic and Optoelectronic Devices Laboratory, Department of Physics, Cochin University of Science and Technology, Kochi, 682022 Kerala India

**Keywords:** ZnO/CuO hierarchical structure, Hydrothermal, Room temperature gas detection, p–n Heterojunction

## Abstract

The development of room temperature gas sensors having response towards a specific gas is attracting researchers nowadays in the field. In the present work, room temperature (29 °C) ethanol sensor based on vertically aligned ZnO nanorods decorated with CuO nanoparticles was successfully fabricated by simple cost effective solution processing. The heterojunction sensor exhibits better sensor parameters compared to pristine ZnO. The response of the heterojunction sensor to 50 ppm ethanol is, at least, 2-fold higher than the response of the ZnO bare sensor. Also the response and recovery time of ZnO/CuO sensor to 50 ppm ethanol are of 9 and 420 s whereas the values are 16 and 510 s respectively for ZnO sensor. The vertical alignment of ZnO nanorods as well as its surface modification by CuO nanoparticles increased the effective surface area of the device and the formation of *p*-CuO/*n*-ZnO junction at the interface are the reasons for the improved performance at room temperature. In addition to ethanol, the fabricated device has the capability to detect the presence of reducing gases like hydrogen sulfide and ammonia at room temperature.

## Introduction

The effective detection and removal of toxic gases in the atmosphere is important for human as well as any living organisms. The uncontrolled release of toxic gases such as CO, H_2_S, NH_3_, CH_3_CH_2_OH, etc. from automobiles, industries, laboratories, etc. cause severe health problems and they may even cause death [1–3]. The use of nanostructured materials for fabricating gas sensors with high sensitivity and selectivity is attracting attention of researchers nowadays because these materials can be easily synthesized and integrated into low cost portable gas detection devices [4, 5]. Among the various nanostructured materials, metal oxide nanostructures belong to the widely accepted category for fabricating gas sensors especially because of their chemical and thermal stability, tunable electrical and optical properties, etc. [6, 7].

Numerous metal oxide nanomaterials such as ZnO, TiO_2_, SnO_2_, WO_3_ etc. [8–11] are commonly used in the field of gas sensing. Nanomaterials are already established in the field of gas sensing especially because of their high sensitivity originated due to their large surface to volume ratio [11]. One dimensional ZnO nanorods are attractive candidates for gas sensor applications because of their increased surface to volume ratio compared to other morphologies of ZnO and most importantly they provide an easy path way for electron transfer. There are several techniques such as doping, forming hierarchical structures, etc. which can be employed to improve the gas sensing properties especially to lower the operating temperature of metal oxide nanostructure based gas sensors. Among the various methods available, forming hierarchical structures using metals (Au, Ag, Pt, Pd, etc.) or metal oxides (CuO, Cu_2_O, TiO_2_, SnO_2_, etc.) [12–14] is an effective way to enhance the various properties of metal oxide gas sensors. Researchers have already found the enhanced gas sensing characteristics of metal oxide/metal oxide hierarchical structures [15–17]. The hierarchical structure can form either p–n, n–n or p–p type semiconductor junctions depending on the nature of the material under consideration. In the present study we have investigated the enhanced gas sensing characteristics of *n*-ZnO/*p*-CuO hierarchical structures. Vertically aligned ZnO nanorods were grown by seed mediated hydrothermal method and CuO nanoparticles were loaded on the surface of ZnO nanorods via simple wet chemical method. ZnO is a well known n-type semiconductor having a direct band gap of 3.37 eV [18]. Various nanostructures of ZnO are used in several application such as photovoltaic [19], gas sensors [20], spintronics [21], etc. CuO is a p-type semiconducting material with a band gap of 1.35 eV which is widely being used in the fields of solar energy conversion [22], gas sensors [23], batteries [24], magnetic storage media [25], transparent electronics etc. *p*-CuO and *n*-ZnO can be combined in different ways to utilize the advantages of p-n heterojunction in gas sensor applications. The improvement in sensing performance of these composites have been attributed to many factors, including electronic effects [26] such as: band bending due to Fermi level equilibration, charge carrier separation, depletion layer manipulation and increased interfacial potential barrier energy. The chemical effects [27] such as decrease in activation energy, targeted catalytic activity and synergistic surface reactions; and geometrical effects [28] such as grain refinement, surface area enhancement, and increased gas accessibility also leads to the improvement in sensing. In addition to achieving better sensor characteristics, minimization of operating temperature and power consumption are the current trends in gas sensor technology. Most of the gas sensors based on metal oxides operate at temperatures above 150 °C which increase the power consumption of the gas sensor. Also the high temperature operation inhibits the use of sensors in explosive environments. In this context the development of room temperature gas sensors with enhanced gas sensing performance have significant importance in the gas sensor industry.

Here, we have grown vertically aligned ZnO nanorods on ITO/glass substrates by a seed mediated hydrothermal method. The growth of ZnO nanorods oriented along c-axial direction by seed mediated hydrothermal method have already reported in literature [29]. ZnO/CuO hierarchical structures were synthesized by depositing CuO nanoparticles on ZnO by a wet chemical method followed by annealing at 250 °C in air. The *n*-ZnO/*p*-CuO heterojunction device was used to detect ethanol, hydrogen sulfide and ammonia at room temperature (29 °C).

## Experimental

### Materials

All the reagents used were analytically pure and used without further purification. Zinc acetate dihydrate (Zn(CH_3_COO)_2_·2H_2_O), sodium hydroxide (NaOH) and copper acetate hydrate (Cu(CO_2_CH_3_)_2_H_2_O) were purchased from fisher scientific. Ammonia solution, isopropyl alcohol and ethanol were purchased from Merck Millipore. De ionized water was obtained from an ultra filter system. ITO/glass substrates were purchased from Sigma Aldrich (surface resistivity 15–25 Ω/sq). The substrates were cleaned by standard cleaning procedure.

### Synthesis and characterization

A thin layer of ZnO seed layer was deposited by immersing the cleaned ITO/glass substrate in a solution containing zinc acetate (0.025 M) and sodium hydroxide (0.05 M) in 100 ml ethanol. The substrate was immersed in the solution for 5 min and the dipping process repeated for 8 times to obtain a uniform ZnO layer over a considerable area of the substrate. In between each dipping process the sample was kept at 80 °C on a hot plate. The annealing of the substrates at the optimized temperature 250 °C in air results in the formation of ZnO nanoparticles. The ITO/glass substrate with ZnO nanoparticle seed layer will act as a lattice matched substrate for the hydrothermal growth of aligned ZnO nanorods. The precursor solution for hydrothermal experiment was prepared by dissolving zinc acetate (0.1 M) and ammonia (25%) in 100 ml de-ionized water. The solution was transferred into a Teflon lined autoclave with the seed layer coated substrate immersed horizontally facing up and kept at 180 °C for 1 h in a laboratory oven. After hydrothermal experiment the samples were taken out and sonicated in iso propyl alcohol for few seconds to remove the unaligned nanorods lying over the vertically aligned nanorods. CuO nanoparticles were deposited by a wet chemical method. 0.05 M copper acetate solution was prepared in ethanol at room temperature and ZnO sample was immersed in the solution for 1 h. After deposition the sample was annealed at 250 °C for 2 h in air to form ZnO/CuO heterostructure.

The crystal phase and crystallinity of ZnO/CuO hierarchical structure was investigated by glancing angle X-ray diffraction taken using PANalytical X’pert PRO high resolution X-ray diffractometer (HRXRD) with CuKα (λ = 1.5418 Å). The detailed microstructure of the samples was analyzed using JEM2100 transmission electron microscopy (TEM) measurements. Raman spectra were recorded using Horiba Jobin–Yvon LABRAM HR Raman spectrometer excited with the 514 nm line of an Ar^+^ laser. The surface morphology of the samples was analyzed using Carl Zeiss field emission scanning electron microscopy (FESEM). The absorption spectra of the samples were recorded using JASCO V-570 spectrophotometer. Room temperature photoluminescence (PL) of the samples were measured using Horiba Jobin–Yvon Fluoromax-3 spectrofluorimeter using Xe lamp as the excitation source. The p–n junction characteristics of the device were studied using Keithley 4200 Semiconductor analyzer.

Gas sensors were fabricated by depositing circular gold electrodes on the top of the samples by thermal evaporation technique. The gas sensing measurements were done in a homemade stainless steel chamber by applying constant voltage. The applied voltage was 1 V for ZnO alone and 8 V for ZnO/CuO structure. Initially we measured the current through the sensor in synthetic air until it reaches a stable value. For all the sensing measurements commercially available high purity sample gases with moisture content less than 2 ppm have been used. Various concentrations of target gas have been injected into the chamber and the corresponding variation in the current through the sample was measured using Keithley source measure unit. After each measurement the chamber opened and samples have been exposed to air to attain the initial resistance. The response of the sensor can be defined as

1 $$ S = \frac{{I_{g} - I_{a} }}{{I_{a} }} $$

where *I*_*g*_ and *I*_*a*_ are the current measured in the presence of the target gas and synthetic air respectively. We have taken *I*_*a*_ as the average value of first 50 points measured in the presence of air which is used for calculating the sensor parameters. The response time and recovery time of the sensor can be defined as the time taken for the sensor to reach 90% and 10% of the maximum response respectively.

## Results and discussion

**Fig. 1:**
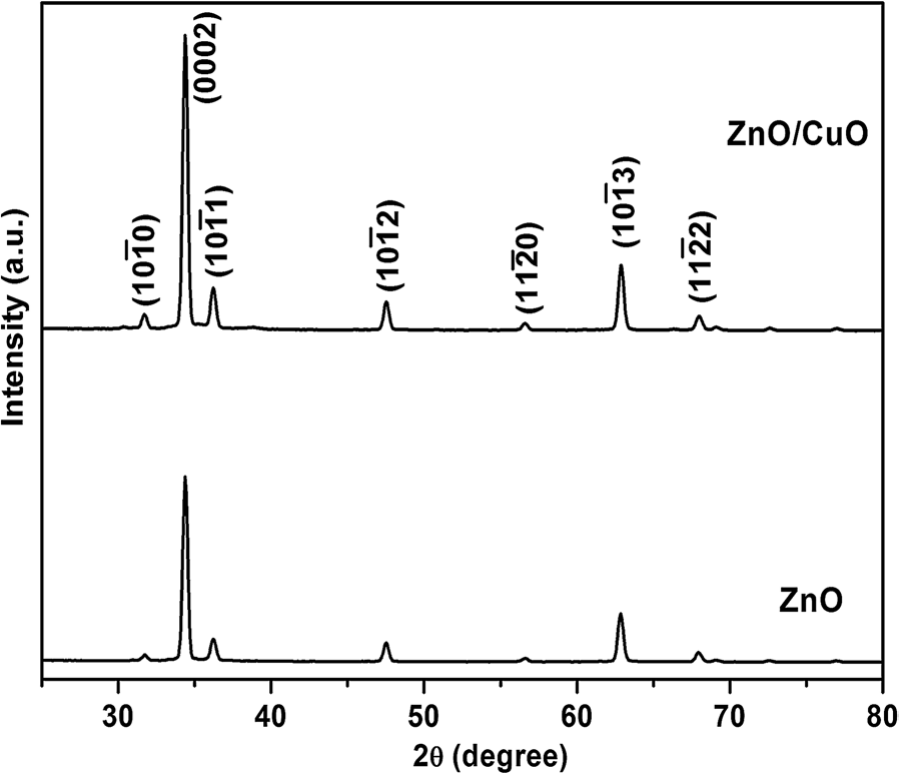
Glancing angle X-ray diffraction pattern of ZnO and ZnO/CuO hierarchical structure

The crystal structure as well as crystallinity of the samples was analyzed using high resolution glancing angle X-ray diffraction shown in Fig. 1. The highly dispersed small CuO nanoparticles were not identified with X-ray diffraction. All the observed diffraction peaks correspond to wurtzite hexagonal ZnO and no peaks corresponding to CuO have been observed in the spectra. The high intensity of the peak along (0002) direction confirms the c-axial growth of ZnO nanorods [30].

**Fig. 2:**
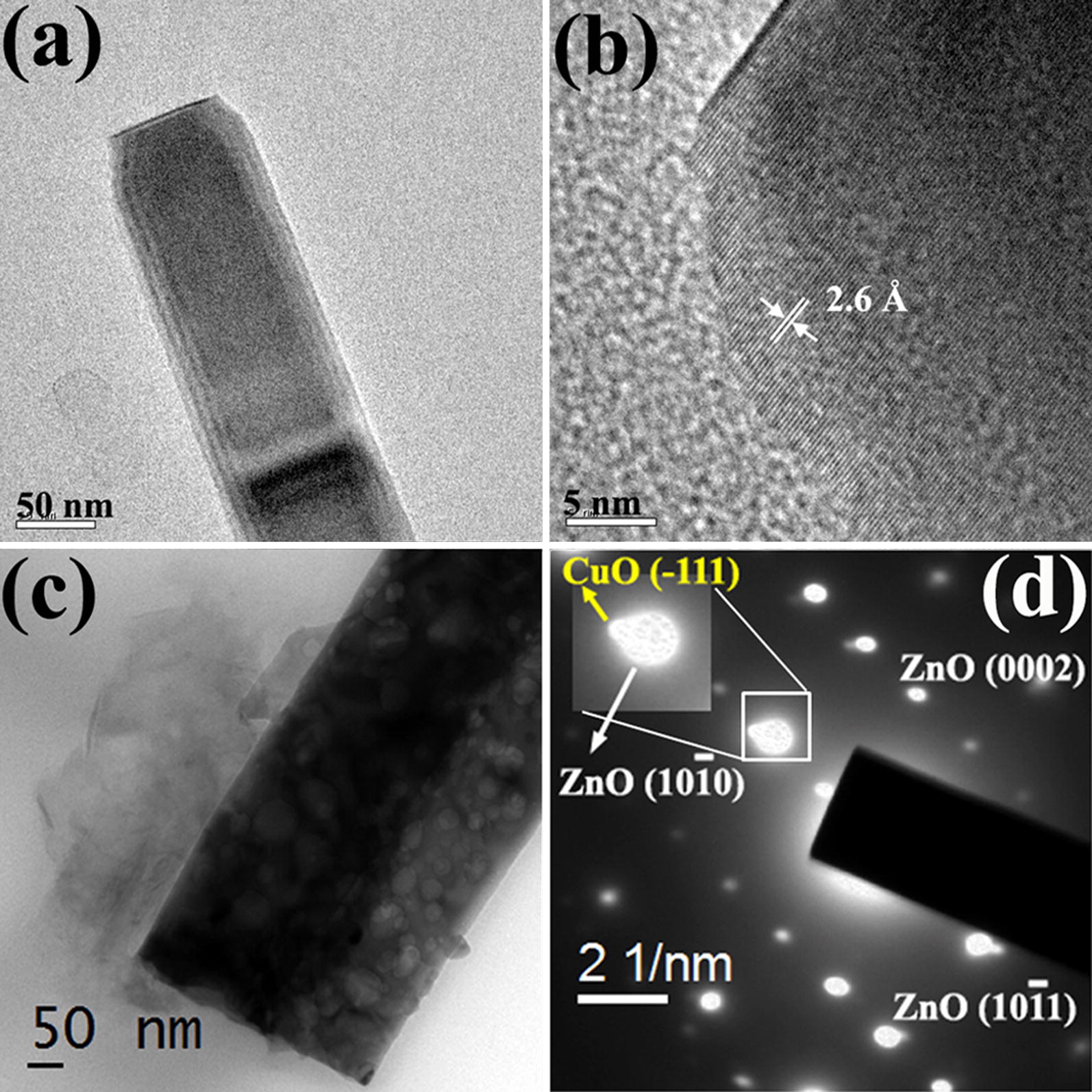
**a** TEM and **b** HRTEM images of ZnO nanorod, **c** TEM image and **d** SAED pattern of ZnO/CuO hierarchical structure

The microstructure of the samples was further analyzed using TEM measurements. The TEM image in Fig. 2a shows the one dimensional morphology of the nanorods and the observed lattice planes in Fig. 2b matches with $\left( {0002} \right)$ plane of ZnO with a lattice spacing of 2.6 Å. The CuO nanoparticles can be seen on the surface of ZnO nanorods in Fig. 2c which make the nanorod surface rough. The presence of bright spots in the SAED pattern in Fig. 2d indicates the crystalline nature of ZnO/CuO structure [30]. In addition to (0002), $\left( {10\bar{1}0} \right)$ and $\left( {10\bar{1}1} \right)$ planes of wurtzite hexagonal ZnO, $\left( {\bar{1} 11} \right)$ lattice plane of monoclinic CuO can also be observed in the SAED pattern confirming the formation of ZnO/CuO hierarchical structures.

**Fig. 3:**
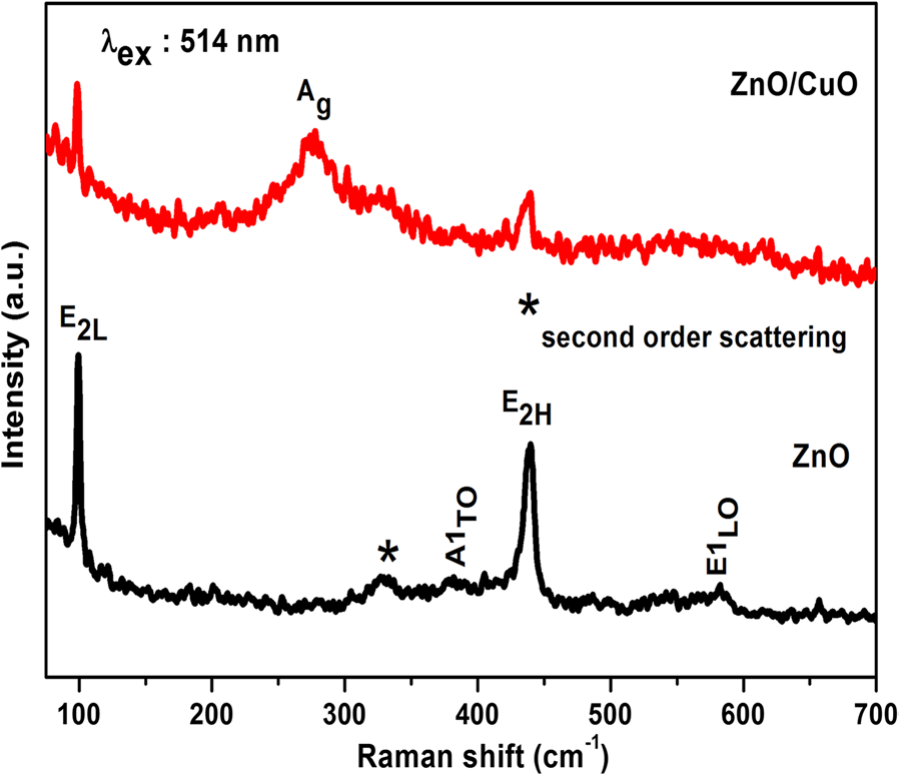
Micro Raman spectra of ZnO and ZnO/CuO hierarchical structure

Micro Raman spectroscopy is a non destructive technique used for analyzing the vibrational properties of materials. The Raman spectra of both ZnO and ZnO/CuO are displayed in Fig. 3. All the observed vibrational modes such as E_2L_ (98 cm^−1^), A1_TO_ (381 cm^−1^), E_2H_ (437 cm^−1^), and E1_LO_ (580 cm^−1^) corresponds to wurtzite hexagonal structure of ZnO. Monoclinic CuO exhibit three Raman active modes (Ag + 2Bg) which are assigned respectively at 278 cm^−1^ (A_g_), 333 cm^−1^ (B_1g_) and 620 cm^−1^ (B_2g_) [31, 32]. Along with the vibrations of ZnO, A_g_ mode corresponding to monoclinic CuO has been observed for ZnO/CuO heterostructure. The Raman vibrations of CuO are highly dependent on the method of preparation and this may be the reason for the absence of B_2g_ vibration. The co-existence of ZnO and CuO Raman modes in the Raman spectra confirms the formation of ZnO/CuO hierarchical structure.

**Fig. 4:**
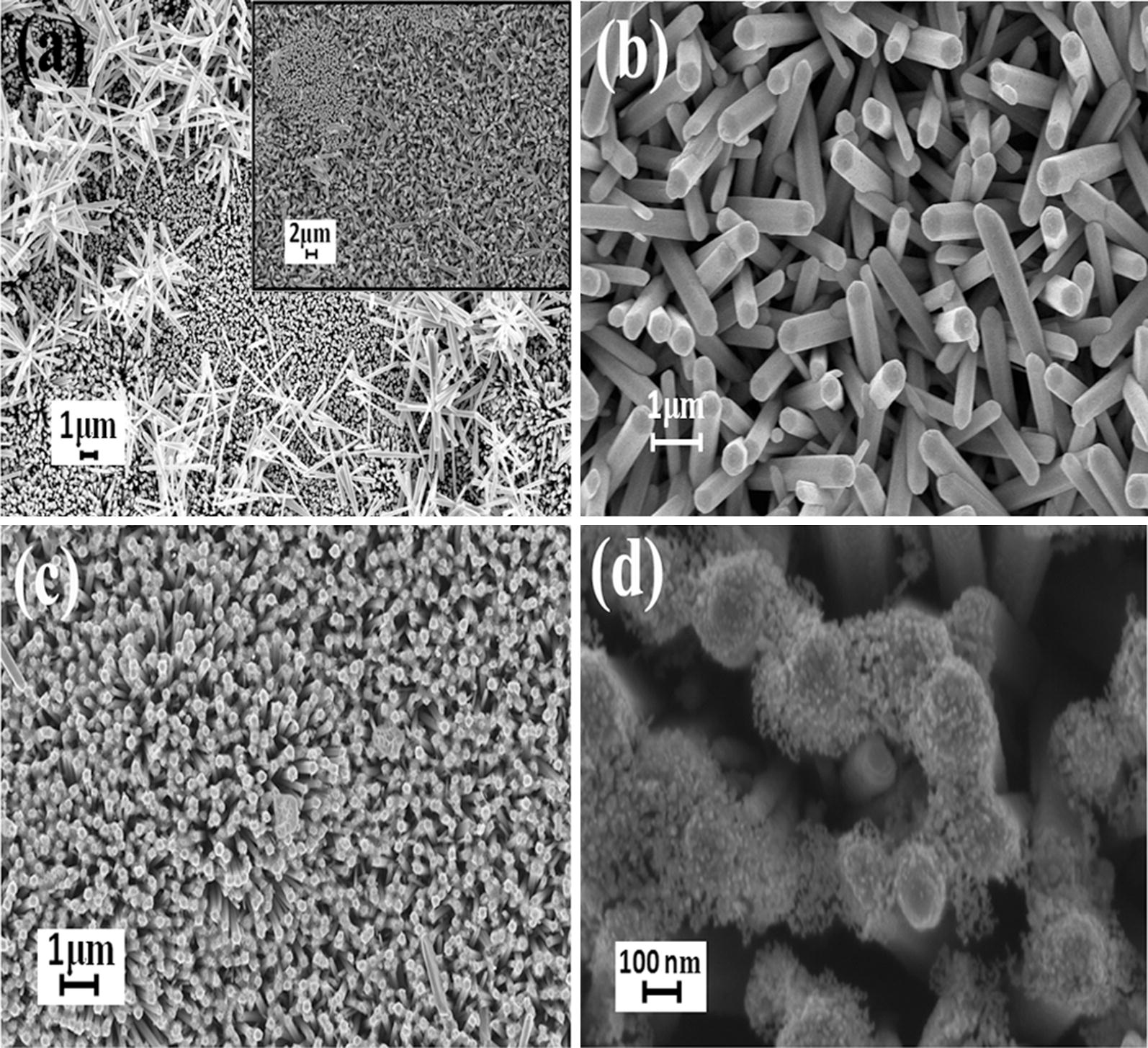
FESEM images of **a** as grown ZnO nanorods (inset shows the image of sonicated sample), **b** magnified view of the sonicated sample, **c** CuO attached ZnO nanorods and **d** magnified view of ZnO/CuO

The surface morphology of all the samples was analyzed using FESEM images depicted in Fig. 4. The vertical alignment of nanorods against the substrate surface forms a porous network which makes the gas diffusion in and out easier [30]. The sonication has effectively removed the unaligned nanorods lying over the vertically aligned nanorods shown in the inset of Fig. 4a. The diameter and length of the nanorods are approximately 95 nm and 2 μm respectively. The presence of CuO on ZnO nanorods can be clearly seen in Fig. 4d. The attachment of CuO increases the interfacial area and correspondingly an enhanced gas sensing behavior can be observed.

**Fig. 5:**
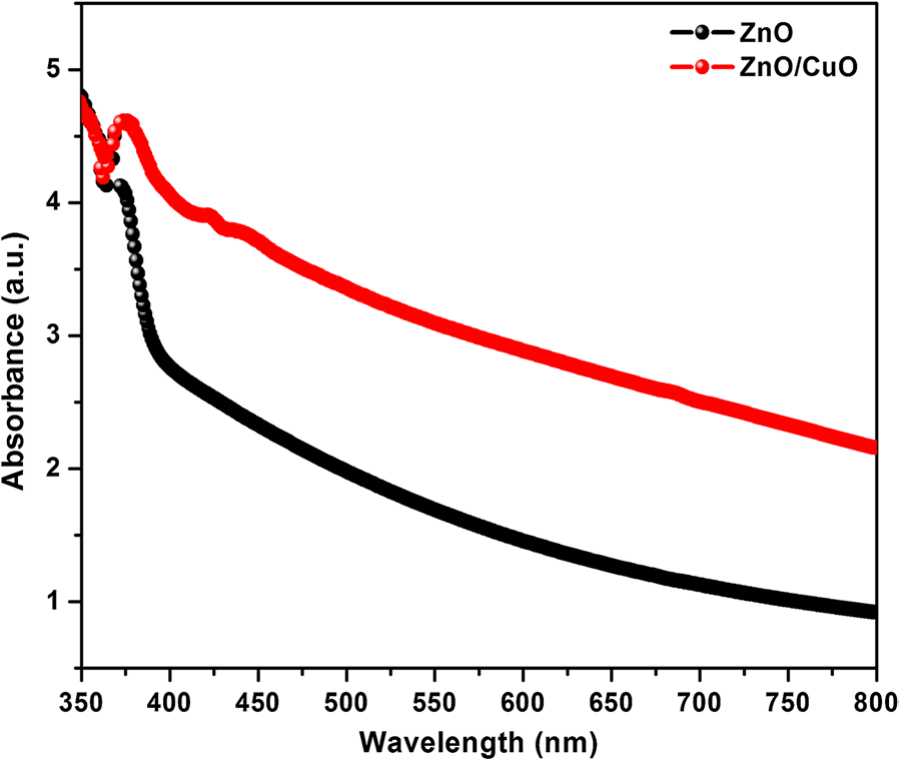
UV–visible absorption spectra of ZnO and ZnO/CuO structures

The UV–visible absorption spectra of ZnO and ZnO/CuO hierarchical structures are shown in Fig. 5. The spectra of pure ZnO nanorods possess an absorption at around 370 nm corresponding to the band gap of ZnO whereas the band gap absorption edge get slightly red shifted to 374 nm in the case of ZnO/CuO hierarchical structure similar to that observed in previous reports [33, 34]. Also the ZnO/CuO sample has a high value of absorbance in the visible region compared to pristine ZnO. These factors confirm the formation of CuO loaded ZnO hierarchical structures.

**Fig. 6:**
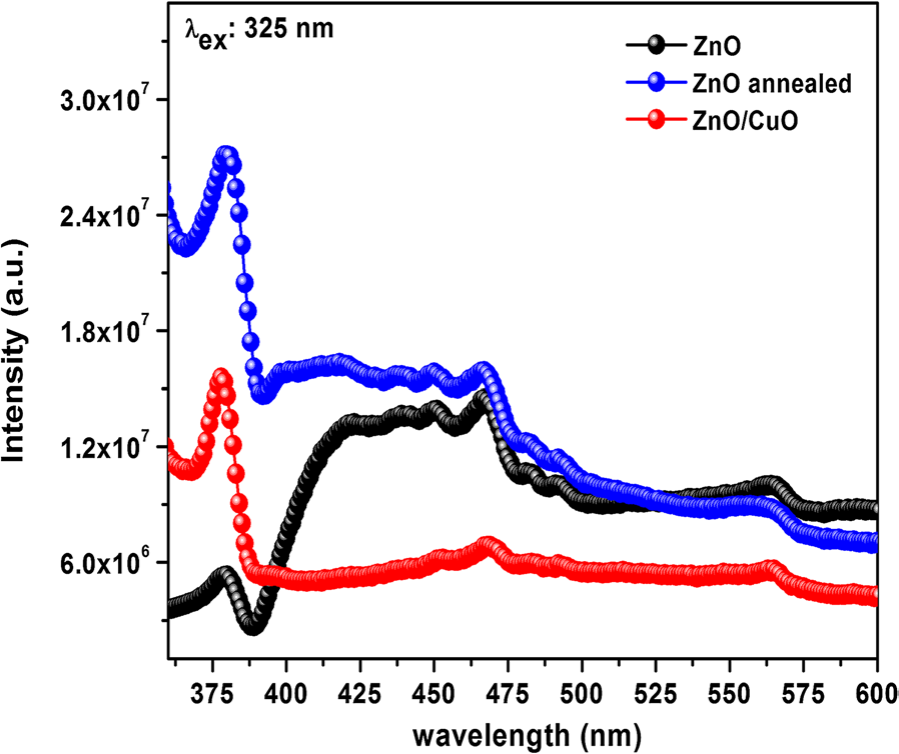
Photoluminescence emission spectra of ZnO and ZnO/CuO heterostructures

The defects such as oxygen vacancies, zinc interstitials, etc. in ZnO nanostructures affects the electronic and surface properties of the semiconductor [35, 36]. The presence of these defect states are in correlation with the performance of a semiconductor gas sensor. Photoluminescence (PL) is a non destructive technique to analyze the defect states in materials. The room temperature PL emission spectra of ZnO and ZnO/CuO heterostructures excited at 325 nm are shown in Fig. 6. For both the samples emissions bands are observed in the UV as well as in the visible region of the electromagnetic spectrum. The UV emission shoulder at 378 nm corresponds to the characteristic emission closely related to the band gap of ZnO. The emission bands in the visible region can be attributed to the transitions between various defect levels within the band gap of ZnO [37, 38]. Oxygen vacancies are one of the important defect states especially in metal oxides which make most of them n-type semiconductors. The emission band at 564 nm in both ZnO and ZnO/CuO samples corresponds to the presence of oxygen vacancies which make them suitable for the fabrication of gas sensors [39] because gas sensing is solely a surface phenomenon which depends mainly on the exposed surface area and the presence of oxygen vacancies in the sensing material. Thus both Raman and PL confirm the presence of considerable amount of oxygen vacancies in ZnO and ZnO/CuO structures. The intensity of defect related emissions got reduced in ZnO/CuO which can be attributed to the formation of *p*-CuO/*n*-ZnO junctions suppressing the recombination of carriers. The increased intensity of band edge emission in ZnO/CuO is due to the annealing of the sample at 250 ^°^C.

**Fig. 7:**
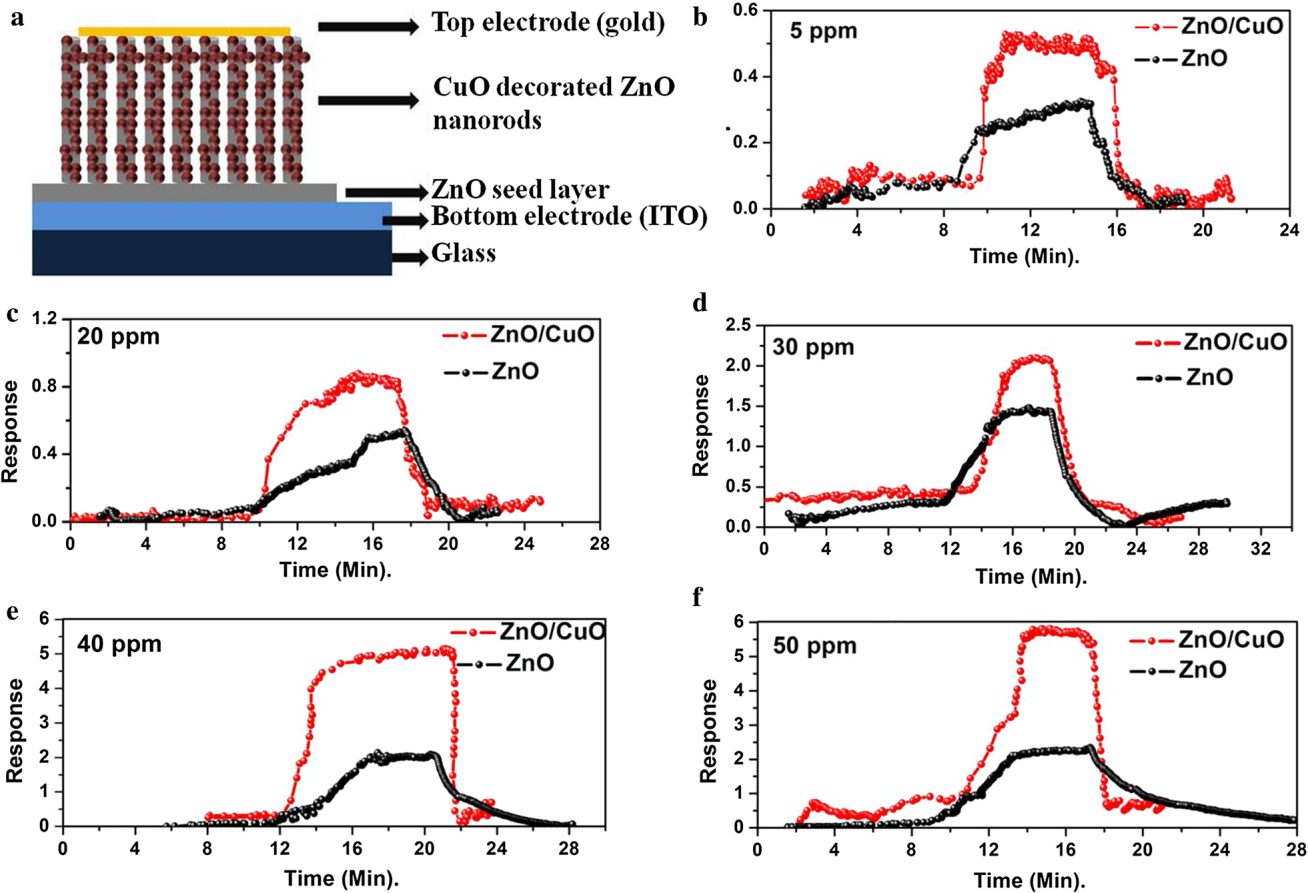
Schematic representation of the **a** device structure and **b**–**f** room temperature ethanol sensing characteristics of ZnO and ZnO/CuO nanostructures

The room temperature (29 °C) ethanol sensing characteristics of ZnO and ZnO/CuO nanostructures were monitored by measuring the change in current upon exposure to different concentrations of the target gas. The response of ZnO and ZnO/CuO to various concentrations of ethanol is shown in Fig. 7. The room temperature response of the sensor increases in ethanol ambient due to the redox reactions taking place between the metal oxide and the target gas which will be discussed later. The room temperature (29 °C) operation of the sensor prevents the grain growth in the sensing material and also reduces the power consumption of the device. Both ZnO and ZnO/CuO samples exhibit very good response to ethanol even for 5 ppm concentration at room temperature. The response of both the sensors increases with increase in concentration of the target gas. Compared to pristine ZnO, ZnO/CuO exhibit improved response values for all the concentrations used in the present study. The vertical alignment as well as the attachment of CuO nanoparticles on ZnO nanorod surface increases the exposed surface area of the sensor contributing to the enhanced sensing characteristics. More importantly the p–n junctions formed at the interface of *n*-ZnO and *p*-CuO significantly improve the gas sensor performance. The detailed mechanism of the heterojunction device will be discussed later.

**Fig. 8:**
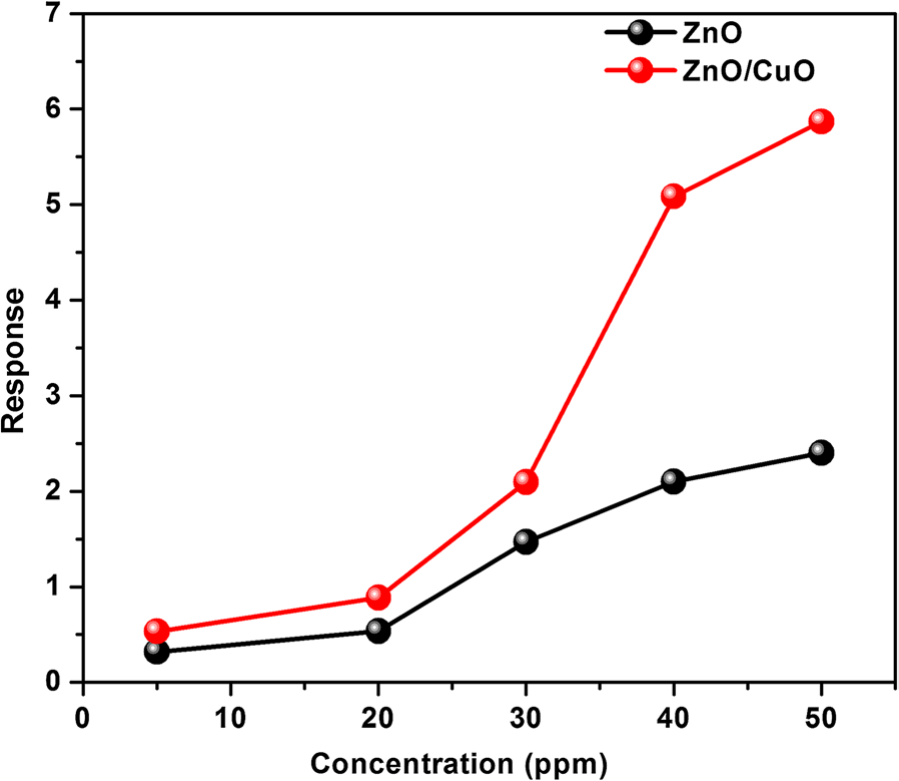
Comparison of ethanol response of ZnO and ZnO/CuO structures

**Fig. 9:**
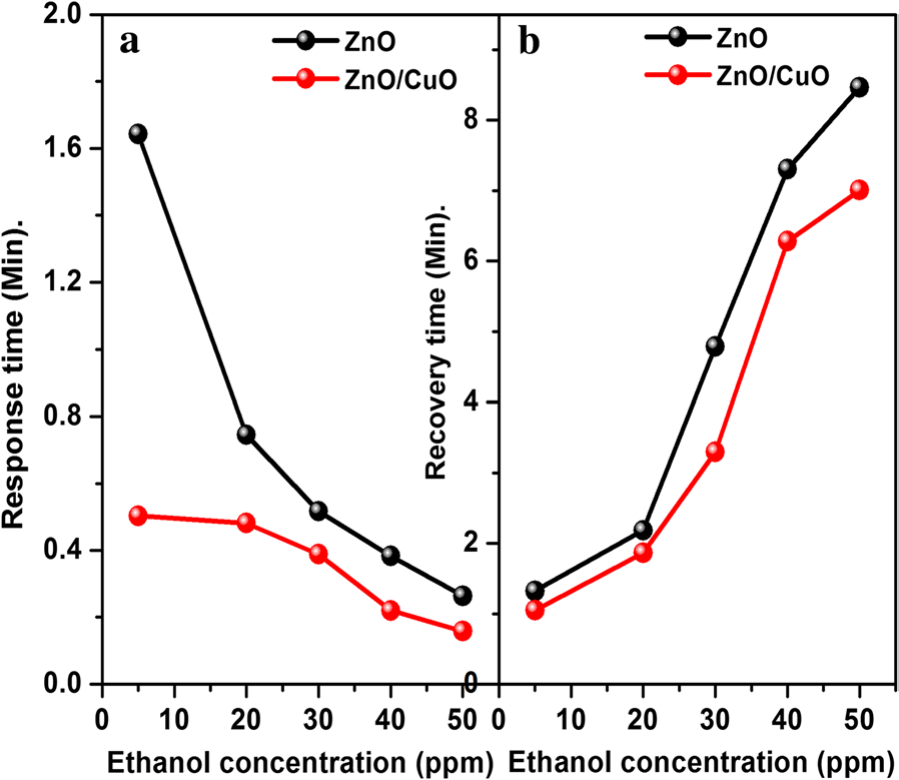
**a** Response and **b** recovery time of ZnO and ZnO/CuO structures to ethanol

Figure 8 shows that the response of ZnO/CuO structure is higher than the response of ZnO for all target gas concentrations. The response and recovery time of the fabricated sensors are depicted in Fig. 9. It can be seen that the response time decreases with increase in concentration whereas the recovery time increases with increase in target gas concentration. This can be attributed to the number of molecules having minimum required energy for the reaction increases at high concentrations hence more and more target gas molecules react with adsorbed oxygen ions resulting in faster change in resistance. Whereas the adsorption takes place slowly at low concentrations due to the lower coverage of gas molecules hence the change in resistance also takes place slowly. The significance of the present work is that even at room temperature both the sensors respond to 5 ppm ethanol gas within less than 100 s. The response time calculated is 98 and 30 s for ZnO and ZnO/CuO respectively and almost complete desorption of the target gas takes place especially at lower concentrations within a few minutes. A good sensor should have high value of response and low value of response time. The complete solution processed p-n heterojunction sensor fabricated in the present study exhibit very good values of gas sensor parameters at room temperature compared to the previous reports [40, 41]. The high value of recovery time of the devices is due to the slow desorption rate of ethanol at room temperature [42]. The incorporation of suitable noble metal additives such as Ag, Au, Pd, Pt, etc. is an effective way to improve the response time of metal oxide based gas sensors [43, 44].

**Fig. 10:**
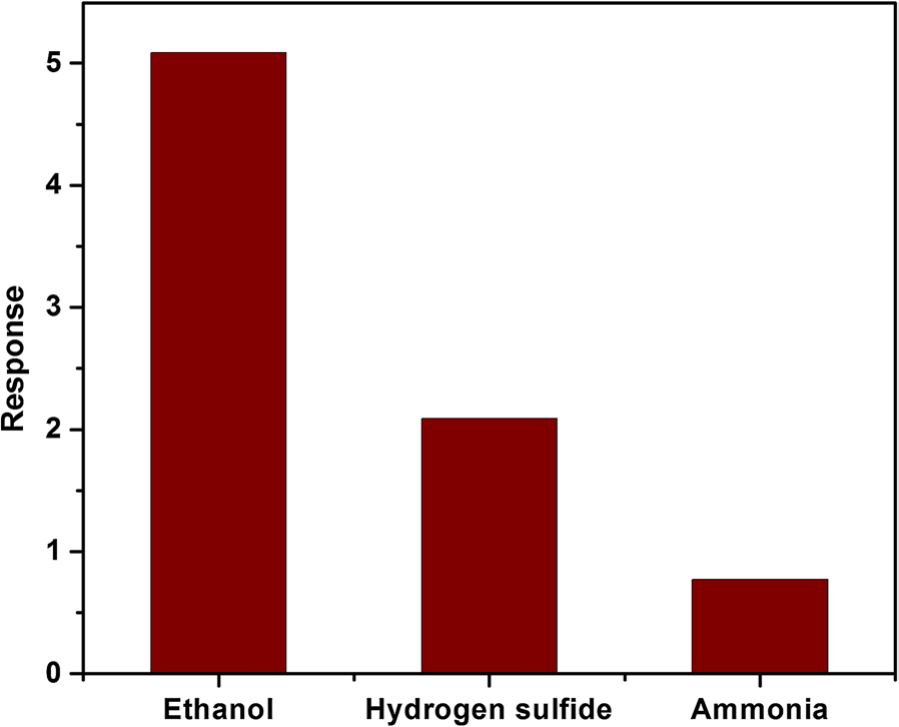
Response of ZnO/CuO hierarchical structure to various reducing gases (40 ppm) at room temperature

The selectivity of ZnO/CuO nanostructure has been studied by testing the response of the device to different types of target gases. Figure 10 shows the response of ZnO/CuO sensor to 40 ppm concentration of ethanol, hydrogen sulfide and ammonia. The response value is 5.08 for ethanol whereas it is 2.091 and 0.772 for hydrogen sulfide and ammonia respectively indicating good selectivity towards ethanol. This is because the electron donating effect of different types of gas molecules is different which depends on the nature of the gas as well as the sensor material.

Table 1 compares the gas sensing characteristics of ZnO/CuO gas sensor with the present work. The simple processing technique and better gas sensing parameters make the fabricated device in the present work a promising candidate for the development of room temperature gas sensors.

**Table 1:** Evaluation of the development of gas sensors based on ZnO/CuO structures

Method of synthesis	Sensor working temperature (°C)	Target gas concentration (ppm)	Response	Response time (s)	Recovery time (s)	References
Hydrothermal	220	Ethanol 100	25.5^a^	6	42	[45]
Hydrothermal	300	Ethanol 100	98.8^b^	7	9	[46]
Solid state reaction	Room temperature	Ethanol 150	2.3^b^	70	88	[41]
Pulsed laser deposition	Room temperature	Hydrogen sulphide 15	78^c^	180	15	[47]
Hydrothermal	Room temperature	Ethanol 5	0.53^d^	30	63	Present work
		50	5.87^d^	9	420	

^a^ $$ S = \frac{{V_{g} \left( {5000\,mV - V_{a} } \right)}}{{V_{a} \left( {5000\,mV - V_{g} } \right)}} $$

^b^ $$ S = \frac{{R_{a} }}{{R_{g} }} $$

^c^ $$ S = \left( {\frac{{I_{g} - I_{a} }}{{I_{a} }}} \right) \times 100 $$

^d^ $$ S = \left( {\frac{{I_{g} - I_{a} }}{{I_{a} }}} \right) $$

CuO hierarchical structure exhibit good response to various reducing gases and the fabricated devices are more selective to ethanol at room temperature (29 °C). The basic gas sensing mechanism of metal oxide semiconductors relies on the interaction between the adsorbed oxygen molecules on the surface of the sensor material and the target gas [5, 7, 48–51]. Generally *O*_2_^−^ at temperature < 100 °C and *O*^−^ and *O*^2−^ at temperature > 100 °C are the dominant oxygen species adsorbed on the semiconductor. The adsorption of oxygen ions on the surface of oxide semiconductor forms an electron depletion region by withdrawing electrons from the conduction band. The interaction between the adsorbed oxygen ions and ethanol gas release electrons back to the semiconductor consequently the depletion layer width and resistance of the semiconductor decreases.

**Fig. 11:**
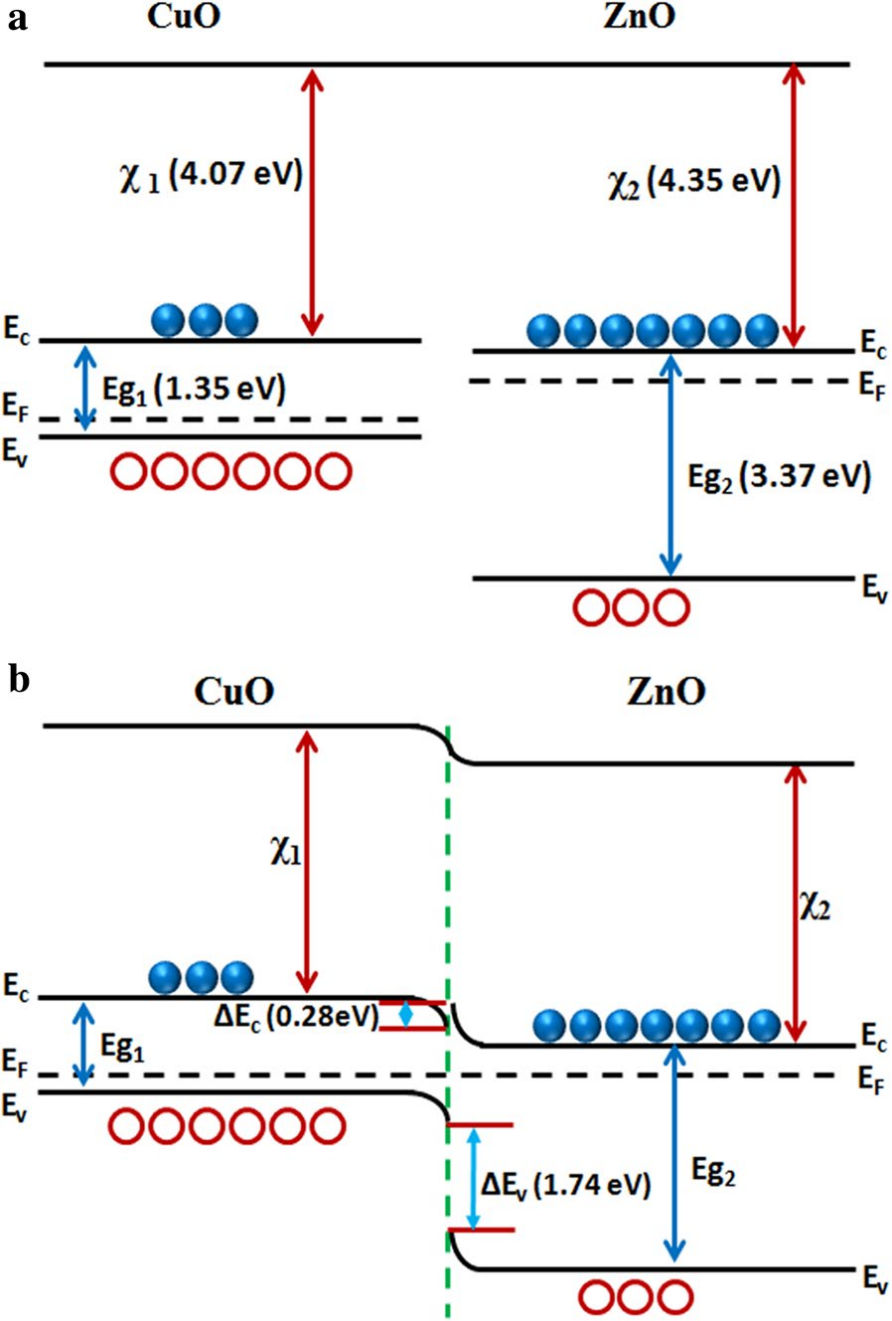
Energy-band diagram of **a** CuO and ZnO and **b** ZnO/CuO heterojunction device at thermal equilibrium

**Fig. 12:**
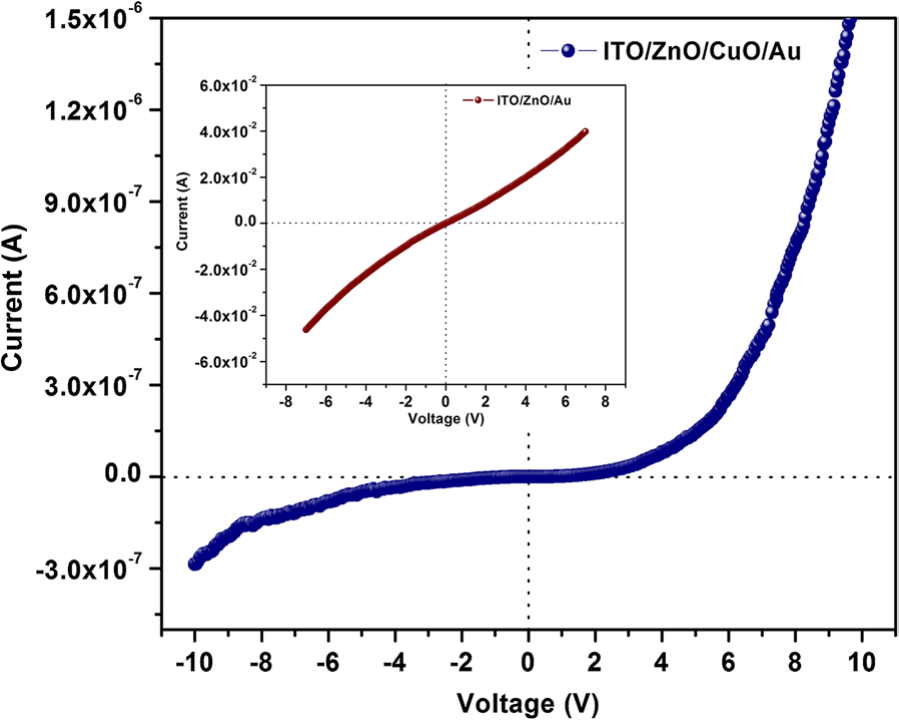
Current–voltage characteristics of ZnO/CuO hierarchical structure (Inset shows the I–V characteristics of ZnO alone

The reasons for the improved sensing behavior of ZnO/CuO hierarchical structures can be attributed to 1) increased number of active sites for gas adsorption [52] and 2) the formation of p-n heterojunctions at the interface of *p*-CuO and *n*-ZnO [15, 53, 54]. The high surface to volume ratio of nanorods and the presence of CuO nanoparticles together increased the number of gas adsorption sites. Also the nanogaps in the nanorod array make more target gas molecules to penetrate into the sensing material. The schematic energy band diagram of *p*-CuO/*n*-ZnO heterojunction at thermal equilibrium is shown in Fig. 11b. Generally oxygen deficient ZnO exhibit n-type and oxygen excess CuO exhibit p-type conductivity. When there is a difference in Fermi energy between the materials forming a junction, electrons from the higher energy will flow across the interface to the lower energy until the Fermi energies have equilibrated. This leads to the formation of a depletion region and a potential barrier at the interface. The presence of a number of p–n junctions at the interface results in a remarkable increase in the resistance of the heterostructure compared to pristine ZnO or CuO. The total resistance of the heterostructure will be contributed by the depletion layer on ZnO, accumulation layer on CuO and the depletion region at the junction and the increased resistance is clear from the current–voltage (I–V) characteristics in Fig. 12. Because of this increased resistance of the heterojunction we have chosen a voltage (8 V) higher than the turn on voltage of the diode for sensing measurements. The response time and recovery time of the sensor depends on the activation energy for gas adsorption and desorption and rate of gas desorption. Both these factors depend on the morphology and composition of the sensing material. In the present work the one dimensional morphology of ZnO as well as the attachment of CuO nanoparticles increase the number of adsorption sites for oxygen and may decrease the activation energy for gas adsorption and desorption processes at room temperature resulting in enhanced gas sensing performance.

In the energy band diagram shown in Fig. 11, *E*_*g*1_ (1.35 eV), *χ*_1_ (4.07 eV) and *E*_*g*2_ (3.37 eV), *χ*_2_ (4.35 eV) represents band gaps and electron affinities [16, 23, 46, 55] of CuO and ZnO respectively. The barrier height of conduction band $\left( {\Delta E_{C} = \chi_{2} - \chi_{1} } \right)$ and valence band $\left[ {\Delta E_{V} = \left( { E_{g2} - E_{g1} } \right) - \Delta E_{C} } \right]$ at the p–n junction were 0.28 eV and 1.74 eV respectively. The generated free electrons on adsorption of ethanol gas in ZnO can easily transport through the p–n junction due to the low value of $\Delta E_{C}$ and at the same time the holes in CuO will accumulate at the valence band of p–CuO due to the large value of $\Delta E_{V}$. At low temperatures the dissociation of ethanol into aldehyde (CH_3_CHO) and H_2_O are prominent than the formation of CO_2_ and H_2_O [41, 56, 57]. At room temperature the dehydrogenation of ethanol molecules generate *OH*^−^ ions (breaking of C-O bond) and [*CH*_3_*CH*_2_*O*]^−^ ions (breaking of O–H bond) due to the lower bond breaking energy of C-O and O–H bonds. Ethanol vapor can be easily attached to metal oxide surfaces in the form of dehydrogenated ionic fragment [*CH*_3_*CH*_2_*O*]^−^ through the interaction of adsorbed oxygen on metal oxide surfaces represented by the Eq. (2). Also at the interface of ZnO/CuO junction ethanol molecules react with holes in CuO [51, 58–60] followed by the Eq. (3).

2 $$ {\text{CH}}_{3} {\text{CH}}_{2} {\text{OH}}_{{(g)}}  + {\text{O}}_{{2(ads)}}^{ - }  \to \left\{ \begin{gathered}   \left[ {{\text{CH}}_{3} {\text{CH}}_{2} {\text{O}}} \right]_{{(ads)}}^{ - }  + {\text{OH}}_{{(ads)}}^{ - }  \hfill \\   {\text{CH}}_{3} {\text{CHO}} + {\text{H}}_{2} {\text{O}} + e^{ - }  \hfill \\  \end{gathered}  \right. $$

3 $$ {\text{CH}}_{3} {\text{CH}}_{2} {\text{OH}}_{(g)} + 2h^{ + } + e^{ - } + {\text{O}}_{2(ads)}^{ - } \to {\text{CH}}_{3} {\text{CHO}} + {\text{H}}_{2} {\text{O}} + e^{ - } $$

These reactions release free electrons resulting in the enhanced room temperature gas sensing performance of *p*-CuO/*n*-ZnO heterojunction device.

## Conclusions

ZnO/CuO heterojunction gas sensor has been successfully fabricated by low temperature solution processing and its room temperature (29 °C) response to various reducing gases has been investigated. Working at room temperature, the response to ethanol gas of the fabricated device is higher than to hydrogen sulfide or ammonia gases. All the gas sensor parameters have been improved by the incorporation of CuO nanoparticles on ZnO nanorods. The easy preparation technique and room temperature gas sensing of the samples will make the practical use of these devices with reduced power consumption a reality.
